# Supplementary material for: Patterns of Intron Gain and Loss in Fungi
Source: PLoS Biol. 2004 Nov 30;2(12):e422. doi: 10.1371/journal.pbio.0020422 (PMC532390; doi:10.1371/journal.pbio.0020422)
Supplement: Table S1 — Also available at http://genes.mit.edu/NielsenEtAl/. (4.3 MB ZIP). [file pbio.0020422.st001.zip › NielsenEtAl/html/918.html]

AN1066.1.NCU02623.1.MG01210.1.FG00663.1


```
 CLUSTAL W (1.82) Multiple Sequence Alignments - Introns Inserted


Sequence 1: MG01210.1	253 aa
Sequence 2: FG00663.1	232 aa
Sequence 3: NCU02623.1	241 aa
Sequence 4: AN1066.1	236 aa
Alignment Length: 254 aa
Number Identitical Residues: 79 aa
Alignment Score (without introns) 4517


MG01210.1 	MRVISKEEEAAHYS~ATIKGGLVGGSVGLALGLAGVVGASKRYAAFRSLTLPFRAFLVTS
NCU02623.1	MKVISKEEEAAHYR~EVVKGGLIGGTGGLIVGLGGVMLGMKRYQFVRQLSLPFRAFLVTS
FG00663.1 	MKILTKEEEEAHYS~AVLKGGFIGGSLGLTVGLGGVFLASRRYPAFRGLTLPFRTFLVTS
AN1066.1  	MKLLTKEEEDAHYR2EVLKGGSFGTVLGLIGGYAGVLAASRRYHTIRNLTLPMKAFLVTS
          	*::::**** ***   .:*** .*   **  * .**. . :**  .* *:**:::*****

MG01210.1 	SGTFGAITVAERYSIAFAKQQDPMNFYKDESTRVLEEAR-QQASQMQRLKDWGRENRYTI
NCU02623.1	AGTFGAIILAEQYSVTFQRAKDPMLNYIEAGKKKHEQERLANVTSTQRFMEWGRENRYSI
FG00663.1 	AGTFGAIINADRWSMDFQKKQNPMNFYEDQTQRAQQIVR-ENQTAMERFMEYGKENRYSI
AN1066.1  	SGTFVGIIGADRSSRSFEAAQNQNRQWYENREERLRAQELRGMSFSDRALAWAREERYTI
          	:*** .*  *:: *  *   ::    : :   .  .  .    :  :*   :.:*:**:*

MG01210.1 	VFASWLASMGVALAAVGKDKYLTTAQKLVQARVYAQGLTLAVLIATAVFETADAKSGRGR
NCU02623.1	VFASWIAAMGAAMAIVNRNKYLSASQKLVQARVYAQGLTLAVLIATAAFETADAKSGKGR
FG00663.1 	VFVSWLASMGVAFAMVSRS-PMNTANKVVQARVYAQGLTLAVLIISAIFEVNDAKKGEGR
AN1066.1  	VFSTWVASMIGSFALVGRNPYLSGPQKLVQARVYAQGATLAVLIASAAFEISERRRVRR-
          	** :*:*:*  ::* *.:.  :. .:*:********* ****** :* **  : :  .  

MG01210.1 	WQTVMVVDPNDPEHKHLIEKKIHKEEYEGQDLWMD1MVAAEEKRIANQKK~VREEAAAKV
NCU02623.1	WETVMVVDPNDPTHQHLIEKKVKKEDYEGQNLWQD1MVAAEEKRLAHKKA~QEKQQQEKA
FG00663.1 	WQTVMVVDPNDPEHKRLIEKKIHKEEYEGQDLWKD1MVAAEERRLAEKKQ~AQGSKA---
AN1066.1  	-----ELDAKSASAK--ADSKLSNNTGEDTELWKD~MVAAEEQRLKNKHH2EQEQEQKSE
          	      :*.:..  :   :.*: ::  *. :** * ******:*: .::   . .   . 

MG01210.1 	NGEAKKENSGKTEAASA
NCU02623.1	SASA-------------
FG00663.1 	-----------------
AN1066.1  	EKKEKQK----------
          	. . ...
```
